# Current Status and Future Perspectives of Antibody–Drug Conjugates in Hormone Receptor-Positive Breast Cancer

**DOI:** 10.3390/cancers16101801

**Published:** 2024-05-08

**Authors:** Maria Grammoustianou, Foteinos-Ioannis Dimitrakopoulos, Angelos Koutras

**Affiliations:** 1 Oncology Department, Sotiria General Hospital, 115 27 Athens, Greece; maria.grammoustianou@gustaveroussy.fr; 2 Breast Cancer Survivorship Research Group, Gustave Roussy, 94805 Villejuif, France; 3 Division of Oncology, Department of Medicine, University Hospital, Medical School, University of Patras, 265 04 Patras, Greece; fodimitrakopoulos@upatras.gr

**Keywords:** antibody–drug conjugates, ADCs, hormone receptor-positive breast cancer, HER2-low breast cancer, HER2-negative breast cancer, endocrine resistance, trastuzumab deruxtecan, sacituzumab govitecan, TROP2

## Abstract

**Simple Summary:**

Advanced hormone receptor-positive (HR+) breast cancer remains a significant clinical challenge despite novel treatment options. The standard therapeutic choice for advanced HR+ breast cancer includes endocrine therapy in combination with cyclin-dependent kinase 4/6 (CDK4/6) inhibitors. However, most of the patients eventually develop endocrine resistance leading to disease progression. Antibody–drug conjugates (ADCs) are a promising class of targeted therapeutic agents that selectively deliver highly potent cytotoxic drugs to cancer cells. This review describes their mechanism of action, preclinical and safety data as well as their indications and future perspectives in the treatment of advanced HR+ breast cancer.

**Abstract:**

Breast cancer is the most common cancer type in women. The vast majority of breast cancer patients have hormone receptor-positive (HR+) tumors. In advanced HR+ breast cancer, the combination of endocrine therapy with cyclin-dependent kinase 4/6 (CDK4/6) inhibitors is considered the standard of care in the front-line setting. Nevertheless, resistance to hormonal therapy and CDK4/6 inhibitors eventually occurs, leading to progression of the disease. Antibody–drug conjugates (ADCs) comprise a promising therapeutic choice with significant efficacy in patients with HR+ breast cancer, which is resistant to endocrine treatment. ADCs typically consist of a cytotoxic payload attached by a linker to a monoclonal antibody that targets a specific tumor-associated antigen, offering the advantage of a more selective delivery of chemotherapy to cancer cells. In this review, we focus on the ADC mechanisms of action, their toxicity profile and therapeutic uses as well as on related biomarkers and future perspectives in advanced HR+ breast cancer.

## 1. Introduction

Breast cancer is the most common cancer type in women, and hormone receptor-positive (HR+) breast tumors are the subtype most frequently diagnosed, counting for approximately 80% of all breast cancer cases [1]. Endocrine therapy in combination with CDK4/6 inhibitors constitutes the mainstay of treatment in metastatic HR+ disease, associated with a meaningful survival benefit in both postmenopausal and premenopausal women [2,3,4,5,6,7,8], even for those with a visceral crisis [9]. In the case of the failure of first-line endocrine treatment, the sequential use of hormone therapies remains the backbone of metastatic HR+ breast cancer management. However, during the last decade, the therapeutic landscape for advanced, endocrine-resistant breast cancer has changed with the incorporation of novel targeted therapies including phosphatidylinositol-3-kinase/Akt and the mammalian target of rapamycin (PIK3CA/AKT/mTOR) signaling pathway inhibitors [10,11,12], next-generation selective estrogen receptor (ER) degraders (SERDs) [13,14,15] and poly ADP ribose polymerase (PARP) inhibitors [16,17], as well as novel treatment approaches, such as ER proteolysis-targeting chimeras (PROTACs) [18] or immunotherapy [19,20,21,22], which are currently being evaluated as monotherapies or in different combinations. Until recently, subsequent therapeutic choices for endocrine-resistant metastatic breast cancer were limited to sequential single-agent chemotherapy, usually associated with low response rates [23,24,25].

The vast majority of HR+ breast tumors are characterized as human epidermal growth factor receptor 2 (HER2) negative; however, approximately 60% of them are defined as HER2 low, characterized by low levels of HER2 expression with immunohistochemistry (IHC) expression 1+ or 2+ and negative in situ hybridization (ISH) for HER2 gene amplification [26]. Historically, the HER2 status was categorized as either positive or negative, but advances in the understanding of the underlying molecular mechanisms shed light on the heterogeneity of HER2-negative disease. This group consists of tumors with different expression levels of HER2, including HER2 low, which still can be targetable by antibody–drug conjugates [27,28]. ADCs represent a rapidly developing group of anti-cancer therapy consisting of a monoclonal antibody (mAb), specifically directed against tumor-associated antigens on the cancer cell surface, and a chemotherapy payload linked to the mAb through a linker. Although the first ADC was approved for HER2-overexpressing breast tumors, preclinical data demonstrated activity of ADCs even in tumors with lower HER2 expression levels, regardless of the hormone receptor status [28]. Thereafter, numerous other ADCs were developed, targeting different antigens on the breast cancer cell surface. ADCs have already changed the therapeutic algorithm in metastatic breast cancer, and ongoing clinical trials test their clinical value in early stage disease either as a monotherapy or in combination with other groups of drugs, such as immunotherapy or targeted therapies. 

## 2. Antibody–Drug Conjugates

### 2.1. Mechanism of Action 

ADCs are composed of a cytotoxic agent attached by a linker to a monoclonal antibody. The antibody binds the target on the surface of the cancer cell and then the ADC is internalized into the cell by endocytosis. As a result of lysosomal enzyme cleavage, the payload is released from the mAb and exerts its cytotoxic activity. The clinical efficacy and toxicity profile of ADCs are affected by each of these components [29,30,31,32,33,34].

Optimal antigen selection and antibody formulation can lead to more selective payload delivery with less toxicity. This could be achieved by choosing tumor-specific targets with high density on the cancer cell surface and taking into account their expression on normal tissue cells in order to minimize on-target/off-target toxicity and achieve an acceptable therapeutic window for ADC applications. Currently, more than 50 antigens have been used as targets for the development of ADCs, including, but not limited, to HER2 and trophoblast cell surface antigen 2 (TROP2) [31,35,36,37,38]. Heterogeneous antigen expression could be partly overcome thanks to the bystander effect, which is induced either by the cytotoxic payload release from the mAb before the ADC is internalized into the cancer cell or after the payload has been released intracellularly due to its high transmembrane permeability [31,39,40]. 

The linker formulation defines the drug-to-antibody ratio (DAR) and pharmacokinetic properties of ADCs. The DAR refers to the amount of cytotoxic drug attached to the antibody. Thus, a higher DAR increases the antitumor efficacy even with lower expression of the targeted antigen on the cancer cell surface. Moreover, the cleavable or non-cleavable formulation of the linker regulates the payload release rate and ADC solubility in circulation, thus determining its potency and toxicity profile. Cleavable linkers are designed to conditionally respond to the tumor microenvironment or intracellular environment, such as low pH or proteolysis, while in the case of non-cleavable linkers, the payload is released only after lysosomal degradation of the antibody [39,40].

The cytotoxic payloads are highly efficient chemotherapeutic agents that are approximately 100- to 1000-fold more potent than conventional chemotherapy. Therefore, they are very toxic to be administered freely, but their toxicities can be minimized by conjugating them to a tumor-specific antibody. There are two major types of ADC payloads: tubulin inhibitors (e.g., MMAE, MMAF and DM1) and DNA-damaging agents (e.g., calicheamicin, SN-38 and DXd) [31].

### 2.2. ADCs in HR+ Breast Cancer Available in Clinical Practice

#### 2.2.1. Trastuzumab Deruxtecan 

T-Dxd is a HER2-targeted humanized IgG1 monoclonal antibody, which consists of trastuzumab covalently linked to the DNA topoisomerase I inhibitor deruxtecan by a cleavable tetrapeptide linker. HER2 is a tyrosine kinase receptor that promotes the proliferation, survival and invasiveness of cancer cells upon homodimerization or heterodimerization with other partners of the human epidermal growth factor receptor family. It is a biomarker related to aggressive disease and poor prognosis compared to HER2-negative breast cancers [27]. T-Dxd binds to the extracellular domain of the HER2 receptor on the cancer cell surface, thus being internalized into the cancer cell, and releases its cytotoxic payload intracellularly after enzymatic cleavage from its linker, leading to DNA damage and cell apoptosis. A high drug-to-antibody ratio (8:1) increases the amount of payload delivered to HER2-expressing cells, while its high transmembrane permeability potentiates the bystander cytotoxic effect on proximal tumor cells, regardless of their HER2 expression status. Deruxtecan has a short half-life in plasma, thus reducing the systemic circulation time and off-target effects on normal cells, while the high stability of the linker in plasma allows for the selective cleavage of the ADC into cancerous cells [41]. 

Apart from breast cancer, T-Dxd has been evaluated in many HER2-positive tumors, such as gastric, lung and biliary tract cancers. In the multicohort DS8201-A-J101 study, including patients with HER2-positive advanced solid tumors, meaningful objective response rates (ORRs) were observed in most sub-cohorts, especially in gastric, non-small-cell lung and breast cancers, including a separate cohort for advanced pretreated HER2-low breast tumors [42]. In 2019, based on the results of the DESTINY-Breast01 trial, the FDA granted accelerated approval to T-Dxd in metastatic or unresectable HER2-positive breast cancer patients previously treated with at least two anti-HER2-based regimens [43]. The findings from the phase III, randomized DESTINY-Breast02 trial confirmed the robust activity and safety of T-Dxd in heavily pretreated metastatic HER2-positive patients resistant to T-DM1, proving that a newer ADC can surpass this resistance [44]. In the DESTINY-Breast03 trial, T-Dxd compared with T-DM1 showed the longest reported median progression-free survival (PFS) and a significant increase in OS in patients previously treated with trastuzumab and a taxane, establishing T-Dxd as a second-line treatment in this setting [45]. 

As far as it concerns HR+ breast cancer, there is evidence supporting the use of T-Dxd also in this subgroup. According to the DESTINY-Breast04 study, patients with advanced HR+, HER2-low breast cancer can benefit from T-Dxd [46]. This study randomly assigned 557 HER2-low metastatic breast cancer patients, who had been treated with one or two previous lines of chemotherapy, to receive trastuzumab deruxtecan (5.4 mg/kg, every 21 days) or treatment of the physician’s choice (TPC). The primary end point of the trial was PFS in the HR+ cohort and key secondary end points were PFS in all comers (regardless of HR status) and overall survival (OS) in HR+ and all comers. The vast majority of patients (88.7%) had HR+ tumors, whereas the remaining 11.3% of the study population had HR-negative disease. All HR+ patients had previously received hormonal therapy (a median of two lines) and the vast majority of them had previously received a CDK4/6 inhibitor (70%). According to the recently reported secondary interim analysis (median follow-up of 32 months), the efficacy and safety profile of T-Dxd were consistent with the results of the primary analysis across all key subgroups, regardless of previous CDK4/6 inhibitor administration or HER2 status (IHC 1+ or 2+/ISH−). The median PFS in the HR+ cohort was 9.6 months for the T-Dxd arm and 4.2 months in the standard chemotherapy arm (HR 0.37), and the median OS was 23.9 months and 17.6 months, respectively (HR 0.69). For all patients, the median PFS was 8.8 months versus 4.2 months (HR 0.36) for T-Dxd and the standard chemotherapy arm, while the median OS was 22.9 months vs. 16.8 months, respectively (HR 0.69). No new safety signals were observed [46,47]. 

Regarding the safety profile, T-Dxd was associated with a range of AEs across all grades, with the most frequently reported being nausea, fatigue and alopecia. However, grade 3 or higher side effects were reported in a lower percentage of patients receiving T-Dxd as compared to those treated with the physician’s choice of chemotherapy (52.6% vs. 67.4%). Neutropenia, anemia and fatigue were the most frequent grade 3 or higher AEs observed in the T-Dxd group. The incidence of interstitial lung disease or pneumonitis in patients who received T-Dxd was 12.1%, primarily of grade 1 or 2, whereas 0.8% had grade 5 events [46].

Moreover, recent data support the activity of T-Dxd in breast cancer patients with central nervous system (CNS) involvement. Evidence from the DESTINY-Breast03 study showed that the confirmed CNS ORR was 63.8% for T-DXd versus 33.3% for T-DM1. However, it should be pointed out that only patients with stable brain metastases were eligible in this trial and the type of local interventions as well as brain lesion radiotherapy status were not available [48]. A recent pooled analysis from the DESTINY-Breast01-02-03 trials of T-Dxd in patients with HER2-positive metastatic breast cancer having both treated and untreated brain metastases (BMs) at baseline (n = 148) reported an intracranial objective response rate (IC-ORR; i.e., CR and PR in brain lesions) in 45.2% and 45.5% of the patients with treated and untreated brain metastases, respectively. The intracranial duration of the response and CNS-progression-free survival rates were substantially prolonged compared to the comparator arm for patients with treated BMs (IC-DoR 12.3 vs. 11 months, and CNS-PFS 12.3 vs. 8.7 months) and untreated BMs (IC-DoR 17.5 vs. 2.8 months, and CNS-PFS 18.5 vs. 4.0 months) [49]. Additionally, according to recently reported data from the phase 2 DAISY trial, investigating the T-Dxd potency in HER2-overexpressing, HER2-low and HER2-negative pretreated metastatic breast cancer patients with clinically inactive brain metastases at baseline (n = 24), T-Dxd elicited a best objective response rate (BOR) of 62.5%. A confirmed BOR was achieved for 91.7%, 30% and 50% of HER2-positive (n = 12), HER2-low (n = 10) and HER2-negative breast cancer patients (n = 2), respectively [50]. The results from these studies suggest the promising efficacy of T-Dxd in patients with brain metastases that warrants further investigation. 

Moreover, DEBBRAH is an ongoing multicohort, phase II study, recruiting pretreated metastatic HER2-positive and HER2-low breast cancer patients with either stable or untreated or progressing brain metastases and/or leptomeningeal carcinomatosis. The results from both the HER2-positive and HER2-low groups demonstrated a high IC-ORR of T-Dxd. In HER2-low cases with asymptomatic brain metastases and progressing brain lesions after local treatment, the IC-ORR was 66.7% and 33.3%, respectively. Notably, the overall IC-ORR in patients with active brain metastases was 46.2%, indicating the promising intracranial activity of T-Dxd, with manageable toxicity [51].

#### 2.2.2. Sacituzumab Govitecan 

TROP2 is a pan-epithelial cancer antigen, which is overexpressed in all subtypes of breast cancer. TROP2 is less expressed on normal tissues, thus representing an attractive target for ADCs [52]. Sacituzumab govitecan is the first-in-class TROP2-targeted ADC that combines a humanized anti-trophoblast cell surface antigen-2 antibody with SN-38, a cytotoxic metabolite of the topoisomerase 1 inhibitor irinotecan, through a cleavable linker. SG is also characterized by a high drug-to-antibody ratio (7.6:1). The results from the ASCENT phase III trial have established SG as the standard of care for metastatic triple-negative breast cancer in the second or subsequent lines of therapy. This study demonstrated that the use of SG was associated with an absolute increase in the median OS from 6.7 months to 12.1 months, compared to a single-agent chemotherapy of the physician’s choice (HR 0.48; *p* < 0.001) [53,54,55]. 

TROPiCS-02 was a randomized, open-label, phase 3 trial, which included 543 pretreated patients with endocrine-resistant HR+, HER2-negative metastatic breast cancer who had received at least one endocrine therapy, a taxane and a CDK4/6 inhibitor in any setting and more than two lines of chemotherapy in an advanced setting [56]. The patients were randomized in a 1:1 fashion to receive either SG (10 mg/kg IV d1 and 8, every 21 days) or the treatment of the physician’s choice. The primary end point was PFS, whereas the OS by the HER2 IHC status was evaluated in an exploratory analysis. In the final OS analysis (data cutoff December 2022), SG demonstrated its superiority in OS over standard chemotherapy. The median OS was 14.5 vs. 11.2 months, favoring SG (HR 0.79; *p* = 0.01). The OS rates at 12, 18 and 24 months were 60.9%, 39.2% and 25.6% for SG and 47.1%, 31.7% and 21.1% for the control arm, respectively [57]. This improvement was independent of the HER2 status by IHC, as the median OS was 13.6 vs. 10.8 months (HR 0.86) for the HER2 IHC 0 group and 15.4 vs. 11.5 months (HR 0.74) in HER2-low cases [58]. 

Because TROP2 is expressed in more than 90% of breast cancers, no biomarker pre-screening was required in the TROPiCS-02 trial of SG, in contrast to the anti-HER2 antibodies for which HER2 screening is mandatory [52]. However, because the TROP2 IHC data were available in about 85% of patients, the SG efficacy was evaluated based on TROP2 expression. This analysis showed that there was a probability of decreased activity with decreased TROP2 levels; however, there was no clear level at which there was a better treatment effect for SG. Membrane TROP2 expression was determined on primary or metastatic archival tumor tissue by IHC and expressed as a histochemical score (H-score) at a range of 0–300. The H-score was ≥100 in 58% of patients and <100 in 42%. The median PFS for an H-score ≥ 100 was 6.4 months with SG and 4.1 months with standard chemotherapy (HR 0.60), whereas for an H-score < 100, it was 5.3 months and 4.0 months, respectively (HR 0.77). An overall survival benefit was observed with SG over the control arm in all the TROP2 H-score subgroups (H-score < 100, median OS 14·6 months vs. 11·3 months; and H-score ≥ 100, median OS 14·4 months vs. 11·2 months), including TROP2 very low expression tumors with an H-score ≤ 10 in which the median OS was 17·6 months vs. 12·3 months, respectively (HR 0.61) [56,57]. 

This is mainly attributed to the high affinity of the antibody and to the SN-38 payload release in the tumor microenvironment without internalization or enzymatic cleavage from the antibody, leading to a bystander effect, regardless of TROP2 expression. Thus, given that SG outperformed chemotherapy in all the prespecified subgroups, immunohistochemical testing for TROP2 expression is not required for selecting patients eligible for treatment with SG [56,57].

The most common AEs included fatigue, hematologic (leukopenia, neutropenia and anemia) and gastrointestinal toxicity (diarrhea and nausea). A dose reduction was required in approximately 30% of patients, mostly due to neutropenia and diarrhea. Drug discontinuation was reported in 6% of patients and was attributed mainly to asthenia and neutropenia. Fatal adverse reactions occurred in 2% of patients [56,57].

## 3. Antibody–Drug Conjugates in Therapeutic Algorithm of HR+ Breast Cancer

In patients with advanced HR+, HER2-negative breast cancer, the combination of endocrine therapy with a CDK4/6 inhibitor is considered the standard of care in first-line therapy, given the ability of this treatment to prolong patients’ PFS [2,3,4,5,6,7,8]. Following the progression of the disease, switching to another hormonal agent is supported by most guidelines, considering that the patient retains some degree of hormonal sensitivity. In this setting, different approaches are available, including single-agent hormonal therapies, as well as combinations of hormonal treatments with other agents. Moreover, recent data supporting the use of CDK4/6 inhibitors in the adjuvant setting makes the therapeutic decisions even more complicated [59]. 

Most of the currently available studies evaluating treatment options following first-line endocrine treatment have used fulvestrant as the control arm. These trials have been associated with poor outcomes for the standard therapy, with a reported PFS in the range of 2–4 months. These suboptimal results clearly indicate the need for the development of more effective options. Therapeutic decisions following progression on first-line treatment with endocrine therapy and CDK4/6 inhibition should be based, whenever this is possible, on a genomic analysis of the patient’s tumor or circulating tumor DNA (ctDNA). This is crucial in order to identify specific groups of patients who could benefit from targeted agents. Given the availability of alpelisib in combination with fulvestrant [10,11], it is important to evaluate for somatic PIK3CA mutations, while detecting PIK3CA/AKT/PTEN alterations could provide a target for capivasertib plus fulvestrant. [60]. Moreover, in patients who relapse during or after the combined hormonal therapy with a CDK4/6 inhibitor, the determination of ESR1 mutations is essential, based on recent data demonstrating the activity of elacestrant in this particular setting [13]. Other novel estrogen receptor-targeted agents, like novel selective estrogen receptor degraders (SERDs), selective estrogen receptor covalent antagonists (SERCAs) or complete estrogen receptor antagonists (CERANs), are also under evaluation [61,62]. In addition, in patients with germline BRCA1/2-mutated tumors, the use of PARP inhibitors such as olaparib and talazoparib has been associated with significantly improved PFS compared to chemotherapy in the OlympiA [16] and EMBRACA trial [17], respectively. The combination of endocrine therapy with the mTOR inhibitor everolimus still remains a viable option for patients without such actionable genomic alterations [12]. 

At the point that the disease becomes refractory to hormonal treatments, a variety of chemotherapy options are used, offering modest benefit to these subpopulations, while specific guidelines do not exist regarding the optimal sequence of these regimens [23,24,25]. Recently, ADCs, such as T-DXd and SG, became available based on the positive results of prospective phase III studies. Currently, there are no clear indications for neither the order nor the efficacy of the sequential use of these ADCs in breast cancer patients. Concerning advanced HR+, HER2-low breast cancer cases, T-DXd has proven its superiority over chemotherapy in second- and third-line treatment according to the DESTINY-Breast04 trial [46]. On the other hand, in the TROPiCS-02 trial, SG showed significant activity in patients with HR+, HER2-negative advanced breast cancer who had been treated with a median of three previous lines of chemotherapy for advanced-stage disease [56]. Moreover, a post hoc subgroup analysis based on the HER2 immunohistochemistry status showed the efficacy of SG in HER2-low as well as in HER2-0 subpopulations [57]. Of note, previous treatment with a CDK4/6 inhibitor was reported in 70% of patients in the DESTINY-Breast04 trial, whereas in the TROPiCS-02 study almost all patients had previous exposure to a CDK4/6 inhibitor. A prespecified subgroup analysis in the HR+ cohort of the DESTINY-Breast04 trial showed a PFS HR 0.55 (95% CI, 0.42–0.74) for CDK4/6 inhibitor-pretreated patients compared to HR 0.42 (95% CI, 0.28–0.64) for CDK4/6 inhibitor-naïve patients, while the median PFS was similar between the two subgroups, i.e., 10.0 months vs. 5.4 months and 11.7 months vs. 5.9 months, respectively [46,47].

**Figure 1:**
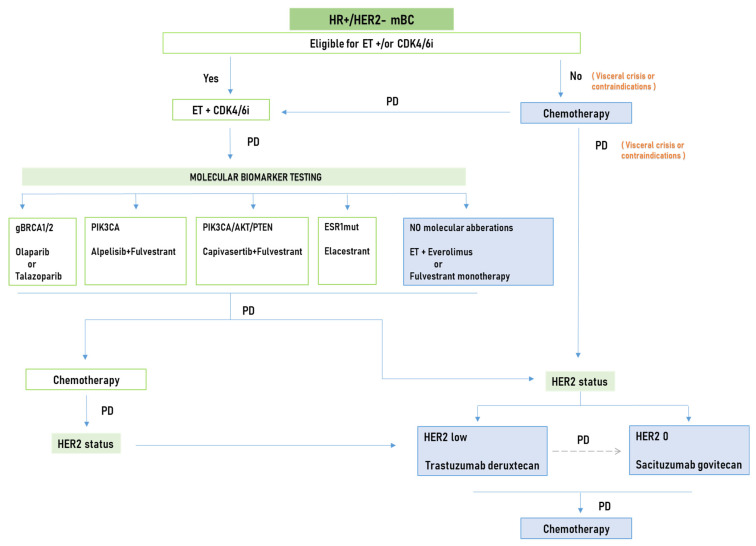
Treatment algorithm for hormone receptor-positive, HER2-negative metastatic breast cancer patients. ET, endocrine therapy; PD, progression of disease.

Considering the available data from these trials, T-DXd is now considered the standard of care for pretreated HR+, HER2-low cases due to its robust clinical activity in a less heavily pretreated population than SG. However, SG is an option especially for HER2 IHC 0 disease, which was not included in the DESTINY-Breast04 trial. On the other hand, the National Comprehensive Cancer Network (NCCN) guidelines suggest that SG can be used in the second-line setting for unresectable locally advanced or metastatic HR+, HER2-negative breast cancer if the patient is not a candidate for T-DXd and can be considered beyond second-line treatment [63] (Figure 1). So far, there are no clinical data that definitely answer if these two ADCs can be effectively used in sequence, as both contain topoisomerase inhibitors as the payload, which potentially leads to cross-resistance. Emerging evidence from real-world studies supporting that the resistance from the sequential use of ADCs is a relevant scenario shows significantly shorter PFS for the subsequently administered ADC, especially if both ADCs share the same target [64,65]. However, given the recent approval of these drugs, the number of evaluated patients that have already received more than one ADC is still small for extracting safe conclusions.

## 4. Future Perspectives 

Numerous other ADCs, with different payloads and/or different targets, are emerging and tested in clinical trials and some of them have already shown promising activity in HR+ metastatic breast cancer. Also, ADCs are evaluated as monotherapy or in combination with other therapeutic agents in an earlier setting in the treatment of HR+ metastatic breast cancer as well as in adjuvant or neoadjuvant settings. Currently, novel ADC strategies are in development, like bispecific ADCs or ADCs with dual payloads or non-cytotoxic payloads, such as immunomodulatory and anti-apoptotic agents.

### 4.1. Hormone Receptor-Positive Metastatic Breast Cancer

#### 4.1.1. Novel HER2-Targeted ADCs

Datopotamab deruxtecan (Dato-DXd) is another anti-TROP2 drug conjugate that is composed of the topoisomerase I inhibitor deruxtecan, linked to a cleavable tetrapeptide linker that makes it more stable in circulation with a longer half-life than SG. Dato-Dxd has shown promising activity in patients with metastatic TNBC and HR+/HER2-negative breast cancers, according to the results of the phase I TROPION-PanTumor01 trial. In this study, an ORR of 32% and a median PFS of 4.4 to 7.3 months were observed. Notably, in topoisomerase I inhibitor-naïve patients, the ORR was 44% [66]. Proceeding from these findings, the phase III TROPION-Breast01 trial aimed to compare the activity of Dato-Dxd to standard chemotherapy in pretreated HR+/HER2-negative advanced breast cancer patients. In the first interim analysis, the ORR was improved in the Dato-Dxd arm compared to the control arm (36.4% versus 22.9%), while the median PFS was 6.9 months and 4.9 months, respectively (HR 0.63). Moreover, there was a trend toward an OS benefit, but the data are not mature yet. The benefit favoring Dato-Dxd was observed in all patient subgroups, regardless of the number of previous lines of therapy or previous CDK4/6 inhibitor use. Importantly, Dato-Dxd was better tolerated compared to standard chemotherapy, associated with fewer grade ≥3 AEs and led to less dose interruptions and reductions. AEs of special interest included oral and ocular defects, predominately oral mucositis / stomatitis and dry eyes that were mostly of grade 1 or 2 and manageable with a very low rate of discontinuation. The drug-related interstitial lung disease rate was 3% with only 1% related to grade ≥3 [67].

Trastuzumab duocarmazine (SYD985) is one more HER2-directed ADC comprised of trastuzumab covalently bound via a linker to the alkylating agent duocarmazine. Trastuzumab duocarmazine has proved its efficacy in pretreated patients with advanced HER2-positive breast cancer in the phase III TULIP study [68]. Accordingly, a phase I trial [NCT02277717], including pretreated, advanced solid tumors with different levels of HER2 expression, indicated activity of trastuzumab duocarmazine among 47 HER2-low cases. Notably, an ORR of 28% was observed among participants with HER2-low, HR+ breast cancer (all partial responses). The most common all-grade AEs were fatigue, conjunctivitis and dry eye [69]. 

Furthermore, disitamab vedotin (RC48-ADC) is a recombinant humanized anti-HER2 mAb conjugated by a cleavable linker to the microtubule inhibitor monomethyl auristatin E (MMAE). The pooled analysis from the phase I C001 CANCER and phase Ib C003 CANCER studies, which examined the RC48-ADC activity in pretreated breast cancer patients with variable HER2 expressions, demonstrated consistent efficacy in both HER2-positive and HER2-low disease with a manageable toxicity profile. In the HER2-low cohort, the ORR was 39.6% (42.9% for IHC 2+/ISH− and 30.8% for IHC 1+) and the median PFS was 5.7 months (6.6 months for IHC 2+/ISH− and 5.5 months for IHC 1+). A grade 1-2 adverse event was related to gastrointestinal, hematologic and sensory neuropathy toxicity, while neutropenia, GGT elevation and fatigue were the most common grade 3 or more treatment-related AEs [70]. An ongoing randomized, phase III study [NCT04400695] will investigate the efficacy and safety of disitamab vedotin in locally advanced or metastatic breast cancer with low HER2 expression [71].

The potential of HER2 mutations as a biomarker for ADCs is also under investigation. Up to 5% of HER2-negative metastatic breast cancers have HER2 mutations, most commonly on exon 20, which predict the response to anti-HER2 tyrosine kinase inhibitors (TKIs) [72]. Interestingly, a recent study reported that within HER2-mutated breast cancers, about 70% of them are hormone receptor positive, suggesting that the emergence of HER2 mutations may represent a mechanism of acquired resistance to endocrine therapy. Therefore, the use of anti-HER2 agents may partially restore the sensitivity of these tumors to endocrine therapy [73,74]. The DESTINY-Lung01 trial evaluated T-Dxd in metastatic HER2-mutant NSCLC patients. The response rate in this study reached 55%, leading to a median PFS of 8.2 months and a median OS of 17.8 months [75]. The ongoing tissue-agnostic DESTINY-PanTumor01 trial is exploring T-DXd activity in patients with solid tumors (other than NSCLC) who have received multiple previous lines of therapy and harbor specific HER2-activating mutations. In this phase II study, the ORRs were 50.0% for breast (n = 20), 20.0% for colorectal (n = 20) and 10.5% for biliary tract (n = 19) cancers [76].

#### 4.1.2. HER3-Targeted ADCs

Human epidermal growth factor receptor 3 (HER3) is overexpressed in breast cancer cells, among many other solid tumors, and is associated with a poor prognosis. Patritumab deruxtecan (U3-1402; HER3-DXd) is a HER3-directed human IgG1 mAb bonded to deruxtecan through a stable, cleavable linker. This agent has demonstrated antitumor activity in preclinical models with a variable HER3 expression status [77,78]. The results from early phase trials have demonstrated the preliminary efficacy of patritumab deruxtecan in HR+/HER2-negative and triple-negative breast cancers. The ICARUS-BREAST01 trial is a phase II study investigating patritumab deruxtecan in endocrine-resistant patients with HR+, HER2-negative metastatic breast cancer following the progression on one line of chemotherapy. This study reported partial responses in 28,6% of patients with an acceptable toxicity profile [79]. 

#### 4.1.3. LIV1-Targeted ADCs

LIV-1 is an estrogen-regulated protein expressed in a number of solid tumors, such as prostate, ovarian and uterine cancer and melanoma, as well as in breast cancer [80]. The Ladiratuzumab vedotin ADC consists of a LIV-1-targeted mAb linked to the MMAE cytotoxic agent by a proteolytically cleavable linker. In a phase I trial including advanced HR+, HER2-negative and triple-negative breast cancers, ladiratuzumab vedotin achieved a disease control rate of 59% and 64%, respectively [81,82]. 

#### 4.1.4. ADCs in Earlier Setting of Metastatic Disease 

Ongoing prospective clinical trials evaluate the use of ADCs in hormone receptor-positive metastatic breast cancer in earlier line settings. The ASCENT-07 trial [NCT05840211] evaluates SG versus conventional chemotherapy in patients with advanced HR+, HER2-negative breast cancer who have progressed on hormonal treatment (with or without a targeted therapy) and who had not received previous chemotherapy in an advanced setting [83]. 

#### 4.1.5. ADC Combinations 

For ADC activity to be enhanced and resistance mechanisms to be overcome, rational combination strategies will probably be key. Current research efforts, based on preclinical data, are exploring different combinations of ADCs with endocrine therapies, immune checkpoint inhibitors and other targeted agents. The phase I DESTINY-Breast08 trial is a multicohort study investigating T-Dxd combinations with other anti-cancer agents, including durvalumab, anastrozole, fulvestrant or capivasertib, in patients with metastatic HER2-low breast cancer, including hormone receptor-positive breast cancer patients [84,85]. The Saci-IO HR+ phase II trial is evaluating the safety and efficacy of SG with or without pembrolizumab in HR+/HER2-negative metastatic disease [86]. Also, the efficacy of disitamab vedotin is evaluated in the phase III trial ROSY in endocrine-resistant, hormone receptor-positive, HER2-low expression metastatic breast cancer [NCT05904964] (Table 1 and Table 2).

### 4.2. Hormone Receptor-Positive Early Breast Cancer

Ongoing trials are investigating the potential role of ADCs in hormone receptor-positive early breast cancer. SASCIA is an ongoing phase III, randomized trial (NCT04595565) testing the efficacy of SG in the adjuvant setting for high-risk, early stage HER2-negative breast tumors having residual disease after standard neoadjuvant therapy. Hormone receptor-negative patients must have residual invasive disease >ypT1mi, while HR+ patients should have a CPS+EG score ≥ 3 or CPS+EG score 2 and ypN+ to be considered as high risk. The first data from the safety interim analysis showed that sacituzumab govitecan related to more all-grade and grade 3 or higher AEs, all of them manageable with appropriate supportive care, and more dose delays and reductions [87]. 

The phase II TALENT trial (TRIO-US B-12) is aiming to evaluate the efficacy of T-Dxd, with or without endocrine therapy, in a neoadjuvant setting for HR+, HER2-low early breast cancer. The vast majority of participants were stage II (77%, n = 45) and had HER2 expression 1+ (79%, n = 46). The ORR was 75% for the T-Dxd-only arm and 63.2% for the T-Dxd plus anastrazole arm [88].

In the I-SPY 2 trial, ladiratuzumab vedotin evaluated at a neoadjuvant setting in high-risk stage II/III HER2-negative breast cancer patients showed analogous efficacy compared to chemotherapy regarding the pCR rates, with similar side effects and even less peripheral neuropathy [89].

Moreover, in early stage HER2-negative breast cancer, the SOLTI TOT-HER3 window-of-opportunity study demonstrated the activity of single-dose patritumab deruxtecan in treatment-naïve patients, regardless of the HER3 levels at baseline [90] (Table 1 and Table 2).

### 4.3. Precision Medicine in ADCs Era

ADCs are part of the technological advances of precision medicine because they have the unique characteristic of combining cytotoxic chemotherapy and targeted therapy using predefined surface targets to deliver highly potent antitumor agents directly to cancer cells. Antigen expression on the surface of the cell is considered as a continuous variable, thus raising the question of whether antigen levels on the surface of the cell should be prescreened for our decision-making. However, among all the molecules that have been used as targets for this class of drugs, HER2 overexpression and/or amplification remain the only biomarkers for therapeutic decisions regarding ADCs. Thus, a lot of efforts in translational research studies are directed toward the evaluation of the anti-HER2 ADCs activity in relation to the HER2 expression status and identifying resistance mechanisms through a biomarker analysis. The ongoing phase III DESTINY-Breast06 trial [NCT04494425] focusing on HER2-negative, ER and/or PR-positive breast cancer aims to investigate the efficacy of T-Dxd in endocrine-resistant, metastatic HER2-low (IHC 2+/ISH− and IHC 1+), HER2-ultra-low (IHC < 1+ but >0) or HER2 non-expression breast tumors. In addition, DAISY is a phase II study that will investigate the T-Dxd activity and explore HER2 expression patterns among advanced breast tumors with HER2 overexpression, HER2 low or HER2 non-expression. In the prespecified interim analysis, this trial reported ORRs of 70.6%, 37.5% and 29.7%, respectively, indicating a T-DXd efficacy even in HER2 non-expressing tumors. There was a significantly greater uptake of T-DXd in HER2-low cells than in HER2-negative cells (*p* = 0.053), indicating that the activity of T-DXd in these cells is due to the bystander effect [91].

There is also evidence that the activity of T-Dxd is not only dependent on the presence of the HER2 antigen but also on its distribution within the tumor, with higher response rates in clustered HER2-expressing cells than if they are spatially distant. In any case, the fact that T-Dxd is active in HER2 non-expressing tumors indicates that the existing definition of HER2-low disease as well as the available evaluation methods are insufficient [92]. According to a recently published survey of data from the College of American Pathologists and a Yale University-based study regarding the concordance of 18 pathologist reports, there was only 26% concordance among pathologists on IHC scores 0 and 1+, compared with 58% concordance between IHC 2+ and 3+ [93]. The ADCs’ activity may be predicted and classified more accurately with the development of quantitative assays for HER2. In addition, HER2 expression is unstable and could change during treatment. Retesting HER2 expression at relapse, when it is possible, is offering the opportunity of a dynamic tracking approach, with the ultimate goal of enhancing therapeutic opportunities. Observation on liquid biopsies has shown the conversion of HER2-negative circulating tumor cells (CTCs) into HER2-expressing CTCs and vice versa [94,95]. 

Numerous other biomarkers are tested as potential targets for novel ADCs in phase I or II trials in an effort to improve efficacy, minimize toxicity and/or surpass resistance to currently approved ADCs. 

### 4.4. Novel ADC Technologies

ADCs with dual or non-cytotoxic payloads as well as bispecific ADCs (BsADCs) are in development in order to exceed tumor heterogeneity and drug resistance. Dual-payload ADCs incorporate two separate payloads, each with a different mechanism of action, providing a potent cytotoxic response by delivering them in a controlled manner to cancer cells [96]. Alternatives to cytotoxic agents, like immunotoxins, pro-apoptotic payloads and immunomodulatory payloads, are also being investigated as payloads in animal models or phase I clinical trials [97,98,99]. Compared to traditional ADCs, BsADCs target dual antigens or dual epitopes, showing enhanced selectivity, improved internalization and eventually treatment effectiveness while minimizing toxicity [100]. 

Alternative technologies to monoclonal antibodies for constructing ADCs are also being explored, such as Nanobodies and protein scaffolds, like the designed ankyrin-repeat proteins (DARPins). These technologies can offer unique advantages over monoclonal antibodies, such as higher specificity, better stability and a smaller size, which can result in improved drug delivery and therapeutic efficacy. Nanobodies, for example, are single-domain antibodies derived from the heavy-chain-only antibodies found in camelids and can penetrate deep into tissues due to their smaller size [101]. DARPins, on the other hand, are protein scaffolds engineered to bind to specific targets with high affinity and selectivity [102].

## 5. Conclusions 

Antibody–drug conjugates have transformed therapeutic algorithms in breast cancer. Given the fact that more than 60% of HR+ breast tumors are expressing HER2 to some extent, the clinical relevance of HER2-low expression has led to new therapeutic opportunities for patients with HR+ disease. Trastuzumab deruxtecan and SG have proved their efficacy over standard chemotherapy in advanced endocrine-resistant HR+, HER2-low breast cancer after second-line therapy, while SG has shown activity even in HER2 non-expressing tumors. Amplification and/or overexpression of HER2 currently remains the only biomarker for therapeutic decisions regarding HER2-targeted ADCs. However, HER2 heterogeneity is an obstacle that could be partly overcome by the high target affinity and bystander effect of novel ADCs. 

At present, no data are available to guide the decision for selection between T-DXd and SG in hormone receptor-positive, HER2-negative metastatic breast cancer patients. In this population, both agents have demonstrated substantial activity with T-DXd proving its efficacy in a less pretreated population, resulting in a preference for using it early in the treatment process. Hence, for patients with endocrine-resistant HR+, HER2-low disease, T-Dxd is the standard treatment option, although SG could be an alternative option in this setting. Currently, SG is the only ADC approved for HER2-zero metastatic breast cancer, despite some evidence indicating that T-Dxd can potentially be active even in this subset of patients. Concerning the sequential use of ADCs, recently reported real-world data suggest significantly shorter PFS for the subsequently administered ADC. Moreover, numerous novel ADCs, like Datopotamab deruxtecan, are showing promising results in this set of patients, giving more potential options for these patients, but at the same time, it may complicate the choice and the sequence of appropriate ADC for each patient even more in the near future. The optimal initial ADC approach remains unclear and may depend on multiple factors, such as target expression or intrinsic resistance to the payload. Identifying the mechanisms leading to ADC resistance would be essential to guide subsequent therapeutic strategies. Therefore, well-designed randomized clinical trials giving emphasis to translational research as well as real-world data are necessary in order to delineate the optimal sequencing and appropriate patient selection for these agents. Finally, considering that ongoing studies may demonstrate the activity of ADCs in earlier lines of therapy, it is possible that these agents will further challenge the sequence of available treatments, as well as the current algorithm in HR+, HER2-negative breast cancer.

## Figures and Tables

**Table 1:** Phase III clinical trials of ADCs in advanced hormone receptor-positive breast cancer.

Trial	Intervention	Comparator	Patients	Setting	Primary End Points	PFS	OS	ORR	AEs
**METASTATIC BREAST CANCER**									
DESTINY-Breast04 Phase III Randomized Open Label	Trastuzumab deruxtecan	TPC (eribulin, capecitabine, gemcitabine, paclitaxel, nab-paclitaxel)	n = 557 HER2 negative HR positive 88.7% HR negative 11.3%	≥1 previous line of HT +/− CDK4/6i and ≥1 previous chemotherapy lines in the metastatic setting	PFS in HR+ disease	HR positive: 9.6 mo (8.4–10.0) vs. 4.2 mo (3.4–4.9); HR 0.37 (95% CI 0.30–0.46) All patients: 8.8 mo (8.3–9.8) vs. 4.2 mo (3.0–4.5); HR 0.36 (95% CI 0.29–0.45)	HR positive: 23.9 mo (21.7–25.2) vs. 17.6 mo (15.1–20.2); HR 0.69 (95% CI 0.55–0.87) All patients: 22.9 mo (21.2–24.5) vs. 16.8 mo (14.1–19.5); HR 0.64 (95% CI 0.48–0.86)	HR positive: 52.6% (47.0–58.0) vs. 16.3% (11.0–22.8) (95% CI) All patients: 52.3% (47.1–57.4) vs. 16.3% (11.3–22.5) (95% CI)	All grades: 99.5% vs. 98.3% Grade ≥3: 52.6% vs. 67.4%
TROPiCS-02 Phase III Randomized Open Label	Sacituzumab govitecan	TPC (eribulin, vinorelbine, capecitabine, gemcitabine)	n = 543 HR positive HER2 negative n = 217; HER2 low, n = 283)	≥1 previous HT, a taxane + CDK4/6i in any setting and 2–4 previous chemotherapy lines in the metastatic setting	PFS	All patients: 5.5 mo (95% CI 4.2–6.9) vs. 4.0 mo (95% CI 3.0–4.4); HR 0.65 (95% CI 0.53–0.81) Exploratory analysis: HER2 IHC 0: 5.0 vs. 3.4 mo; HR 0.70 (95% CI 0.51–0.98) HER2 low: 5.8 vs. 4.2 mo; HR 0.60 (95% CI 0.44–0.82)	All patients: 14.5 mo (95% CI 13.0–16.0) vs. 11.2 mo (95% CI, 10.2–12.6); HR 0.79 (95% CI 0.65–0.95) Exploratory analysis: HER2 IHC 0: 13.6 vs. 10.8 mo; HR 0.86 (95% CI 0.63–1.13) HER2 low: 15.4 vs. 11.5 mo; HR 0.74 (95% CI 0.57–0.97)	21% versus 14% (95% CI 1.03–2.56), p 0.035	Grade ≥3: 74% vs. 60%
TROPION-Breast01 Phase III Randomized Open Label	Datopotamab deruxtecan	TPC (eribulin, capecitabine, vinorelbine, gemcitabine)	n = 700 HR positive, HER2 negative	Previous HT and 1–2 previous chemotherapy lines in the metastatic setting	PFS, OS	6.9 mo (95% CI 5.7–7.4) vs. 4.9 mo (95% CI 4.2–5.5); HR 0.63 (95% CI 0.53–0.76)	OS data not mature HR 0.84 (95% CI 0.62–1.14)	36.4% vs. 22.9%	All grades: 94% vs. 86% Grade ≥3: 21% vs. 45%
Disitamab vedotin (RC48-ADC) NCT04400695 Phase III Randomized Open Label	Disitamab vedotin	TPC (paclitaxel, docetaxel, vinorelbine, capecitabine)	n = 366 (estimated) HER2 low	Previous HT and 1 to 2 lines of prior chemotherapy lines in metastatic setting	PFS	Not yet reported	Not yet reported	Not yet reported	
DESTINY-Breast06 Phase III Randomized Open Label	Trastuzumab deruxtecan	TPC (capecitabine, paclitaxel, nab-Paclitaxel)	n = 886 HR positive, HER2 low or HER2 IHC > 0 < 1+	≥2 previous lines of HT +/−-targeted therapy in any setting	PFS in HR+, HER2-low disease	Not yet reported	Not yet reported	Not yet reported	
ASCENT-07 Phase III Randomized Open Label	Sacituzumab govitecan	TPC (paclitaxel, nab-paclitaxel, capecitabine)	n = 654 (estimated) HR positive, HER2 negative (HER2 IHC0 or HER2 low)	≥2 previous lines of HT +/−-targeted therapy or recurrence within 6 months of first-line HT +/− CDK4/6i or while on the first 24 months of adjuvant HT with CDK 4/6i	PFS	Not yet reported	Not yet reported	Not yet reported	
**EARLY BREAST CANCER**									
SASCIA Phase III Randomized Open Label	Sacituzumab govitecan	TPC (capecitabine, carboplatin, cisplatin)	n = 1332 (estimated)	ADJUVANT HER2-negative patients with high risk of relapse after standard NACT For HR negative: any residual invasive disease > ypT1mi and/or ypN1> 1 mm For HR-positive disease: a CPS+EG score ≥ 3 or CPS+EG score 2 and ypN+	iDFS	Not yet reported	Not yet reported	Not yet reported	SIA (n = 88) All grades: 100% vs. 86% Grade ≥3: 66.7% vs. 20.9%

AEs: adverse events, CI: confidence interval, HR: hazard ratio, HT: hormonal therapy, iDFS: invasive disease-free survival, NACT: neoadjuvant chemotherapy, ORR: objective response rate, OS: overall survival, PFS: progression-free survival, SIA: safety interim analysis and TPC: treatment of physician’s choice.

**Table 2:** Phase I/II clinical trials of ADCs in advanced and early stage hormone receptor-positive breast cancer.

Trial	Intervention	Patients	Setting	Primary End Point	PFS	OS	ORR	Adverse Events
**METASTATIC BREAST CANCER**								
**DAISY** Phase II Open Label	Trastuzumab deruxtecan	n = 186 Cohort 1 (n = 72) HER2 positive Cohort 2 (n = 74) HER2 low Cohort 3 (n = 40) HER2 non-expressing (IHC0+)	At least 1 previous line of chemotherapy in metastatic setting Additionally: For HER2 positive: previous trastuzumab and TDM-1 For HER2 negative, HR positive: previous ET and CDK4/6i and capecitabine	BOR	Cohort 1 11.1 months (95% CI 8.5–14.4) Cohort 2 6.7 months (95% CI 4.4–8.3) Cohort 3 4.2 months (95% CI 2.0–5.7)	Cohort 1 not reached (95% CI 16.7–not reached) Cohort 2 not reached (95% CI 11.5–not reached) Cohort 3 11.6 months (95% CI 8.3–17.3)	Cohort 1 70.6% (95% CI, 58.3–81) Cohort 2 37.5% (95% CI, 26.4–49.7) Cohort 3 29.7% (95% CI 15.9–47)	All grade: Cohort 1 98.5% Cohort 2 98.6% Cohort 3 89.5% Grade ≥3: Cohort 1 33.8% Cohort 2 39.7% Cohort 3 31.6%
**DEBBRAH** Phase II Open Label	Trastuzumab deruxtecan	Cohorts 1 and 3: HER2 positive Cohort 4: HER2 low Cohorts 2 and 5: HER2 positive and HER2 low	For HER2 positive, previous taxane and at least one HER2-targeted therapy for advanced disease For HER2 low/HR negative, at least one previous chemotherapy regimen For HER2 low/HR+, at least one chemotherapy and one HT in the metastatic setting	Cohort 1: 16 weeks PFS Cohort 2, 3, 4: ORR-IC Cohort 5: OS	No results for HER2 low yet	No results for HER2 low yet	No results for HER2 low yet	Grade ≥ 3: 28.6–52.4%
		Cohort 1: Treated BM stable after local intervention * Cohort 2: Asymptomatic BM without clinical requirement for local intervention * Cohorts 3 and 4: New and/or progressive BM following previous local intervention * Cohort 5: LMC with positive CSF cytology						
**Saci-IO HR+** Phase II Randomized Open Label	Sazcituzumab govitecan vs. Sazcituzumab govitecan + Pembrolizumab	n = 110 (estimated) HER2 negative, HR positive	At least 1 prior HT and ≤1 prior chemotherapy for HR+ metastatic setting	PFS	Not yet reported	Not yet reported	Not yet reported	
**ICARUS-BREAST01** Phase II Open Label	Patritumab deruxtecan (U3-1402) (HER3-DXd)	n = 56 HER2 negative, HR positive	Progression on HT and CDK4/6i (+/−-targeted therapies) and one previous chemotherapy in the metastatic setting	ORR	Not yet reported	Not yet reported	3m-RR 28.6%	All grade fatigue (89.3%), nausea (76.8%) Grade ≥ 3: fatigue (14.0%) Confirmed ILD: 1 Grade1 (1.8%)
**DESTINY-Pantumor01** Phase II Open label	Trastuzumab deruxtecan	n = 102 HER2-mutated advanced solid tumors **EXCEPT** HER2-positive breast, gastric or gastroesophageal junction adenocarcinoma or HER2-mutant NSCLC Breast cancer cohort (n = 20)	Progression following prior treatment or no satisfactory alternative treatment options, including approved second-line therapies in the specific tumor type	ORR	All patients 5.4 months (95% CI 2.7–7.1)	Not yet reported	ORR All patients: 29.4% Breast cancer 50%	Grade ≥ 3: 51% ILD 10.8% (Grade 3 n = 1; Grade 5 n = 2)
**DESTINY-Breast08** Phase Ib Open Label	Trastuzumab deruxtecan combos Module 1: T-DXd + capecitabine Module 2: T-DXd + durvalumab + paclitaxel Module 3: T-DXd + capivasertib Module 4: T-DXd + anastrozole Module 5: T-DXd + fulvestrant	n = 139 HER2 low, HR positive and HR negative	For HR positive: Part 1: At least 1 prior line of HT +/−-targeted therapy and 1 prior line chemotherapy in metastatic setting Part 2: No prior chemotherapy in metastatic setting For HR negative: Part 1: At least 1 prior line of chemotherapy for metastatic setting Part 2: No prior lines of therapy for metastatic setting (Module 2), only 1 prior chemotherapy line for metastatic setting (Modules 1 and 3)	Safety, tolerability	Not yet reported	Not yet reported	Not yet reported	
**Trastuzumab duocarmazine (NCT02277717)** Phase I Open Label	Trastuzumab duocarmazine (SYD985)	n = 185 Part I: solid tumors of any origin Part II: breast, gastric, urothelial and endometrial tumors (HER2 IHC ≥ 1)	Locally advanced or metastatic tumor progressed on standard therapy or no standard therapy exists	Dose-limiting toxicities			BC dose-expansion cohorts: HER2+ (n = 48): ORR 33% (all PR) HER2 low, HR pos (n = 32): ORR 28% (all PR) HER2 low, HR neg (n = 15): ORR 40% (all PR)	All grades: fatigue 33% ocular events 71% conjunctivitis 31% dry eye 31% Grade 3 ocular events: 7%
**Ladiratuzumab vedotin (NCT01969643)** Phase 1 Open Label Dose-Escalation	Ladiratuzumab vedotin (SGN-LIV1A)	Multicohort Part A: Triple-negative disease or HR positive, HER2 negative Part E: HR positive, HER2 negative	Part A: ≥ 2 prior chemotherapy in the metastatic setting and no longer a candidate for hormonal therapy Part E: Chemotherapy-eligible and not considered a candidate for further hormonal therapy. Must have received ≤ 1 non-hormonally directed or chemotherapy in metastatic setting	Incidence of adverse events Dose-limiting toxicities	Not yet reported	Not yet reported	DCR (=CR + PR + SD): HR positive, HER2 negative (n = 17): 59%	Grade ≥ 3: neutropenia (25%) anemia (15%)
**EARLY BREAST CANCER**								
**TRIO-US B-12 TALENT** Phase II Open Label	Trastuzumab deruxtecan +/− anastrazole	n = 33 HER2 low HR positive	NEOADJUVANT Previously untreated, operable, invasive carcinoma of the breast ≥cT2, cN0 or cN1/cN2 provided they are deemed to have operable disease at study entry	Efficacy (pCR rate)			pCR: T-Dxd monotherapy: 1/17 pt had pCR and 2/17 pts had RCB-I (17.6% RCB 0/1) T-Dxd+ Anastrazole: 1/16 pt had RCB-I (6.3%) ORR: 75% in T-Dxd monotherpary vs. 63% in T-Dxd+ anastrazole	
**I-SPY 2** Phase II Open Label	Ladiratuzumab vedotin (SGN-LIV1A) followed by AC	n = 24 HER2 negative HR positive	NEOADJUVANT Previously untreated, operable, invasive carcinoma of the breast at least 1 dimension with ≥ 2.5 cm in largest diameter	pCR rate (95% prob interval)			pCR rate: 0.09 (95% prob interval 0–0.18) Probability SGNLIV1A Superior to Control: 0.15	
**SOLTI TOT-HER3** Phase I Single Group Open Label	Patritumab deruxtecan (HER3-DXd) (U3 1402)	n = 37 HER2 negative HR+ (n = 20) TNBC (n = 17)	NEOADJUVANT Previously untreated, operable, invasive carcinoma of the breast at least 1 dimension with ≥ 1 cm in largest diameter	CelTIL score change			Change in CelTIL**: Overall (*p* = 0.046) TNBC (*p* = 0.016) **But not in HR+ (*p* = 0.793)** ORR by US: Overall 32% TNBC 35% HR+ 30%	All grades 84% Grade ≥ 3: 1 patient No ILD

BM: brain metastases, BOR: best objective response, CelTIL: tumor cellularity and tumor-infiltrating lymphocytes score, CI: confidence interval, CR: complete response, CSF: cerebrospinal fluid, DCR: disease control rate, HR: hazard ratio, HT: hormonal therapy, ILD: interstitial lung disease, LMC: leptomeningeal carcinomatosis, NSCLC: non-small-cell lung cancer, ORR: objective response rate, ORR-IC: intracranial objective response rate, OS: overall survival, pCR: pathologic complete response, PFS: progression-free survival, PR: partial response, RCB: residual cancer burden, RR: response rate, SD: stable disease. * WBRT and/or SRS and or surgery ** ERBB3 levels were not associated with CelTIL change or ORR.
